# Serological evidence of arboviruses and coccidia infecting horses in the Amazonian region of Brazil

**DOI:** 10.1371/journal.pone.0225895

**Published:** 2019-12-12

**Authors:** Fábio Alves Gomes, Ana Maria Jansen, Rosângela Zacarias Machado, Hilda Fátima Jesus Pena, Marcílio Jorge Fumagalli, Angélica Silva, Bruna Farias Alves, André Luiz Rodrigues Roque, Luiz Tadeu Moraes Figueiredo

**Affiliations:** 1 Laboratory of Trypanosomatid Biology, Oswaldo Cruz Institute, FIOCRUZ, Rio de Janeiro, Rio de Janeiro, Brazil; 2 Federal Institute of Education Science and Technology of Roraima, Caracaraí, Roraima, Brazil; 3 School of Agricultural and Veterinary Studies of Jaboticabal, São Paulo State University, Jaboticabal, Brazil; 4 School of Veterinary and Animal Science, Department of Preventive Veterinary and Animal Health, University of São Paulo, São Paulo, Brazil; 5 Virology Research Center, Ribeirão Preto Medical School, University of São Paulo, Ribeirão Preto, Brazil; CEA, FRANCE

## Abstract

**Background:**

Arboviruses and protozoans can cause neurologic disorders in horses. In Brazilian Amazon, several horses presenting signs compatible with disorders caused by these infectious agents have been observed.

**Objective:**

To contribute to the knowledge of this epidemiological picture, we sought to construct a serological diagnostic panel for neurotrophic infectious agents in local horses.

**Material and methods:**

A total of 213 blood samples from horses were collected from 29 farms in three municipalities. Samples were evaluated and considered positive when they met the following criteria: titers ≥ 1:80 with the indirect fluorescent antibody test (IFAT) for apicomplexan protozoans; positive recombinant enzyme-linked immunosorbent assay (ELISA) with subsequent titers ≥ 1:10 by the PRNt for viruses; and detection under direct microscopic examination for *Trypanosoma evansi*.

**Results:**

No horses were found to be infected by *T*. *evansi*, and only two were infected *Toxoplasma gondii* and/or *Neospora* spp. The highest protozoan infection rate was observed for *Sarcocystis neurona* (40.3%; n = 86/213). Among the positive ELISA samples tested by the plaque reduction neutralization test (PRNT_90_), 92% (n = 76/83) were positive for St Louis Encephalitis virus, 43% (n = 6/14) were positive for West Nile virus and 33% (n = 16/48) were positive for Mayaro virus. Eighteen percent (n = 39/213) of horses were co-infected by *S*. *neurona* and at least one arbovirus, particularly SLEV and/or MAYV.

**Conclusion:**

Samples positive for SLEV associated with *S*. *neurona*, including samples from horses that had recovered from neurological signs were frequent, and must be considered when investigating the possible causes of neurological diseases in South Roraima horses.

## Introduction

*Sarcocystis neurona*, *Neospora caninum* and *Toxoplasma gondii* are related coccidians that are reported to cause encephalitis in horses [1]. North and South American opossums, *Didelphis virginiana* and *D*. *albiventris*, respectively, are the known definitive hosts of *S*. *neurona* [2]. Horses become infected by ingesting *S*. *neurona* oocysts or sporocysts [3,4] and some horses may develop equine protozoal myeloencephalitis (EPM), a clinical progressively debilitating neurologic disease that affects the central nervous system [2].

Arboviral infections in humans and animals have been increasing globally with dengue (DENV), West Nile (WNV), Zika (ZIKV), chikungunya (CHIKV), Schmallenberg and bluetongue viruses. This phenomenon has been associated with the increased transport of animals and people worldwide, environmental and climate changes, and human encroachment into natural habitats [5]. Most arbovirus infections are asymptomatic or may be present as a mild acute febrile illness. However, several arboviruses are important human and veterinary etiologic agents that can cause disease of the central nervous system, leading to coma and death [5,6].

In Brazil, there are favorable ecological characteristics (availability of vectors, hosts, and other factors) that support the introduction and maintenance of arboviruses, such as DENV, ZIKV, and CHIKV, with a high impact on public health [7]. There are densely populated cities infested by *Culex* and *Aedes* mosquitoes and ecological changes, such as deforestation due to human settlements can affect the transmission cycles of arboviruses [8].

More than 200 different arboviral species have been isolated in Brazil, including 40 viruses have been associated with human diseases [8]. Many of these viruses belong to two taxonomic families: the *Flaviviridae* (*Flavivirus* genus) and *Togaviridae* (*Alphavirus* genus). Flaviviruses such as St. Louis Encephalitis virus (SLEV) and WNV can infect horses and cause infection of the central nervous system, with encephalomyelitis with ataxia being the most common clinical presentation [9–12]. SLEV was previously isolated from a horse’s brain with neurological symptoms. and is widely distributed in the Americas, from Canada to Argentina. In Brazil, SLEV was first isolated in 1960, from a pool of *Sabethes belisarioi* mosquitoes captured at the Belém-Brasília highway in the Amazonian region. In this same region, studies on the SLEV cycle showed that *Culex declarator* and *Culex coronator* were vectors and wild birds, monkeys, sloths, armadillos and marsupials were virus reservoirs [13–15].

In parts of North America, Europe and Asia, *Culex quinquefasciatus* has already been proven to be a capable vector for the transmission of WNV from birds to horses [9,16,17]. This mosquito species is widespread in the American continents (including the western Amazon region) and is able to colonize both urban and wild areas, similar to those areas encountered in the south of the Roraima state [18–20].

Disease in horses due to Rocio (ROCV) *Flavivirus* and Mayaro (MAYV, *Alphavirus*) has not been described. However, ROCV causes human encephalitis, and MAYV produces human febrile acute illness disease with arthropathy [8,21]. ROCV was isolated from a wild bird, *Zenothrichia capensis* and from the mosquito *Psorophora ferox* [14,22], and MAYV antibodies were found in birds from seven families [23]. Moreover, mammals from different orders (Xenarthra, Marsupiala, Rodentia, Carnivora and Artiodactyla) presented antibodies to MAYV in northern Brazil and French Guiana [24,25].

Between June 2014 and May 2016, in the Amazon biome in the south of Roraima state, North Brazil, 25 horses died after presenting neurological symptoms (*i*.*e*., ataxia, poor motor skills, and torsion and bending in the neck). In addition to the economic impact caused by livestock deaths, the clinical and epidemiological characteristics of such cases suggest the involvement of infectious agents. Almost all horses have close contact with ruminants and considering the absence in the horses of respiratory, ocular or reproductive symptoms such as conjunctivitis, nasal discharge, abortion in the final third of pregnancy or death of neonate foals; or abortion and teratogeny between the ruminants that have contact with horses, we consider that Equine Herpes virus, Equine Arteritis virus and Bunyamwera virus have no relevance in the local epizothiological context. Regarding the encephalitic alphaviruses, we observed that there was a discrepancy in the information obtained in the studied farms about horse’s vaccination status. There was a strong divergence in the information provided by the owners and the farm staff about the vaccination for Eastern Equine Encephalitis virus and Western Equine Encephalitis virus, which could lead to misinterpretation of the results.

We, thus, performed a serological survey of horses from the southern region of the Roraima State to determine their levels of antibodies to some selected arboviruses (SLEV, WNV, ROCV and MAYV) and protozoa (*S*. *neurona*, *Neospora caninum* and *T*. *gondii*), and we performed microscopic blood examination for *Trypanosoma evansi* detection, aiming to construct a serological diagnostic panel for neurotrophic infectious agents in local horses.

## Material and methods

### Horse blood samples

A total of 213 native horses and without history of travelling outside the state of Roraima were enrolled in the serologic survey. The criteria employed to define the farms for the survey were as follows: (i) the horses on the farm had contact with dead or recovered horses from neurological symptoms between 2014/2016 (F1, F9, F11, F12, F14, F16, F17, F18, F19, F23, F24, F25, F26, F27, F28, F29), and (ii) the horses that frequented or dwelled on farms where there was a huge agglomeration of horses from other properties (F2, F3, F4, F5, F6, F7, F8, F10, F13, F15, F20, F21, F22). All farms were located in late-70’s settlements. Horses that participated in the study were apparently healthy and did not have a history of central nervous (CNS) infection, except for four animals that had recovered from a neurological disease.

Horse blood samples were collected by external jugular vein and were stored in 2 flasks, one flask without preservatives that was used for the arbovirus and coccidiosis survey and another containing EDTA that was used for hemoparasite direct testing. The tubes were stored in containers with ice and transported to the field laboratory.

### Parasitological and serological assays

Blood smears were stained by Panótico® (Laborclin, São Paulo, Brazil) and microscopically visualized for hemoparasites. After that, blood was centrifuged in microhematocrit tubes and the buffy coat was directly observed between the glass and cover slide by optical microscopy [26].

An indirect immunofluorescence antibody test (IFAT) was employed as previously described [27] to search for *Toxoplasma gondii*, *Neospora* spp. and *S*.*neurona* infections. The strains used as antigens were RH for *T*.*gondii*, and NC-1 for *Neospora* spp. and SN-138 for *S*.*neurona* [28,29], and a cut-off dilution of 1:80 was used for both parasites. The reactions were revealed by an anti-horse IgG conjugate, with the whole molecule produced in rabbit (Sigma Aldrich, Saint Louis, USA) at a 1:64 dilution following the manufacturer's instructions. The *S*. *neurona* merozoites were grown and maintained in African green monkey (*Cercopithecus aethiops*) kidney cells (*CV-1)* in RPMI media supplemented with 10% fetal bovine serum at 37°C and 5% CO_2_. Supernatants with free merozoites were passed through a sterile 3-μm filter and then centrifuged at 1,500*g*, for 10 minutes at 4°C. The pellet was resuspended in phosphate buffered saline (PBS), centrifuged and washed again with PBS. The concentration of merozoites in the filtrates was determined by counting on a hemocytometer and standardized to a concentration of 10^7^ merozoites/ml. Aliquots of 20 μl of SN-138 merozoites in PBS solution were dispensed to each well of a Teflon-coated antigen slide. Slides were air-dried at room temperature, fixed in 100% methanol, air-dried again, and stored at -22°C. For the assay, serum samples were diluted 1:80 in PBS for screening. Diluted samples were incubated for 30 minutes at 37°C. After three 5-minute washes in PBS, slides were air-dried, fluorescein isothiocyanate-conjugated anti-horse IgG was applied to each well, slides were incubated again, washed, air dried and examined with a fluorescence microscope. Samples found positive with the 1:80 dilution screening were further diluted at two-fold increments to obtain the final titer. Positive and negative horse serum controls were included in each slide.

Arbovirus infections were evaluated in duplicates of horse serum samples by an indirect IgG enzyme-linked immunosorbent assay (ELISA) using anti-horse IgG peroxidase conjugate, whole molecule produced in rabbit (Sigma Aldrich, St. Louis, MO, USA). The reaction was quantified by adding 2,2-azinobis (3-ethylbenzthiazolinesulfonic acid) (ABTS) (KPL, Milford, MA, USA).

The ELISA used recombinant antigens of domain III peptides (rDIII) of the envelope proteins of West Nile virus (WNV), of Saint Louis encephalitis virus (SLEV) and of Rocio virus (ROCV), or a recombinant envelope protein 2 (rE2) of Mayaro virus (MAYV). All antigens were produced in an *Escherichia coli* (*E*. *coli*) system as previously described [30,31]. Mouse hiperymmune sera to WNV, SLEV, ROCV and MAYV were used as positive control, and extracts of *E*. *coli* proteins as negative control. The cut-off value in the assay was calculated as the mean optical density (O.D.) of the negative controls plus three standard deviations. Samples with an average O.D. above the cutoff value were considered positive.

### Plaque reduction neutralization tests (PRNTs)

Serum samples that were positive for only one of the flaviviruses in the ELISA were submitted to a 90% plaque reduction neutralization test (PRNT_90_) [32], in Vero cells with the same virus. For MAYV, all positive samples in the ELISA assay were tested, even though they were also positive for flaviviruses. Viruses used in the PRNT_90_ were WNV NY99, SLEV SpAn 11916, ROCV SpH 34675 and MAYV Be Ar 20290. Serum samples were considered as positive by PRNT_90_ when a serum dilution of 1:10 or greater reduced the viral formation of plaques by at least 90%.

### Statistics

We calculated the correlation between seropositivity rates and: i) contact of horses with dead or recovered horses from neurological symptoms between 2014/2016 and ii) the habit to visit or dwell on farms where are a recurrent huge agglomeration of horses from other properties. We use the chi-square contingency (BioEstat, version 5.0), adopting an α = 0.05 level of significance. These analyses also encompassed the 4 horses that had recovered from neurological disease. Considering that WNV (6 positives), *T*.*gondii* (1 positive) and *Neospora* spp. (2 positives) had a low number of positive samples, these agents were not included in the statistical analyzes.

### Geospatial analysis

The satellite images from study área were provided by the Landsat 8 OLI sensor platform, corresponding to scenes 232/59, 231/59 and 231/60 https://earthexplorer.usgs.gov. The map with the location of the area in South America and the state of Roraima was made with the USGS National Map Viewer ttp://viewer.nationalmap.gov/viewer/.

### Ethical statements

Sample collections and handling procedures were approved by the Animal Ethics Committee of the Instituto Oswaldo Cruz (No. L-009/2017), Brazil, and the University of São Paulo Animal Ethics Committee, Brazil (No 66/2018).

## Results

**Fig 1:**
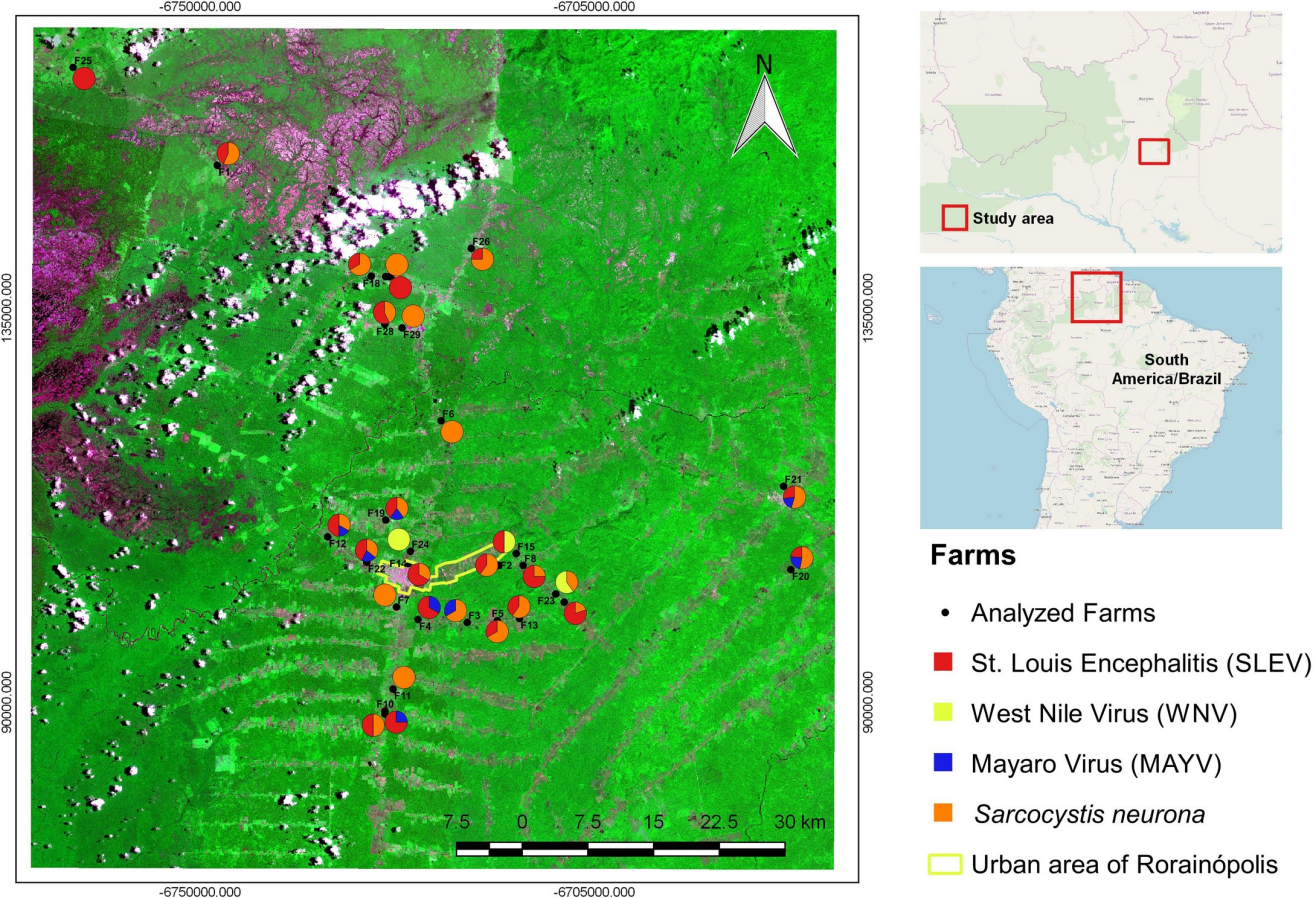
Geographic distribution of the infected horses with viruses and protozoans derived from properties with an epidemiological link or in which cases and/or the death of horses with neurologic disorders occurred. The farms with positive results by plaque reduction neutralization tests (PRNt) for viruses and the indirect immunofluorescence antibody test (IFAT) for *S*. *neurona* in the municipalities of Rorainópolis, Caracaraí and São Luiz, Roraima state, Brazilian Amazon, are shown. The coloured circles represent the proportion of positive results per infectious agents Saint Louis Encephalitis Virus (SLEV), West Nile Virus (WNV), Mayaro virus (MAYV), *Sarcocystis neurona* (*S*. *neurona*).

The highest infection rates observed in horses from southern Roraima state, Brazilian Amazon was for *S*. *neurona* followed by SLEV (Table 1 and Fig 1).

**Table 1:** Results of the PRNT_90_ for viruses and IFAT for protozoans in horses (n = 213) from Roraima state, Brazilian Amazon.

	AGENT / POSITIVES / INFECTION RATE (%)	CONTACT			AGGLOMERATION		
		Yes	No	*P value*	Yes	No	*P value*
PRNT	SLEV / 76 / 35.7	44	32	0.2	34	42	0.4
	MAYV / 16 / 7.5	4	12	0.08	10	6	0.4
	WNV / 6 / 2.8		6		1	5	
IFAT	*Sarcocystis neurona /* 86 / 40.4	44	42	0.9	38	48	0.3
	*Toxoplasma gondii /* 1 / 0.5						
	*Neospora* spp. / 2 / 0.9						

Contact—contact of horses with dead or recovered horses from neurological symptoms; Agglomeration—the habit to visit or dwell on farms where are a recurrent huge agglomeration of horses from other properties. IFAT–imunoflorescence antibody test; PRNT plaque reduction neutralization test,; *P* value–result of chi square test; St. Louis Encephalitis Virus (SLEV), West Nile Virus (WNV), Mayaro virus (MAYV)

### Protozoans

Out of 213 horses 40.4% (N = 86) were positive for coccidiosis: one was positive for the three tested protozoans, one was positive for *Neospora* spp. and *S*. *neurona* and eighty-four (39.4%) were positive for *S*. *neurona* only, with titration ranging 1:80 to 1:5120 by IFAT, being 47 (1:80–1:320), 39 (1:640–1:5120) (S1 Table). For *S*. *neurona*, no difference was observed in positivity regarding contact of horses with dead or recovered horses from neurological symptoms (*p* = 0.9) and the habit to visit or dwell on farms where are a recurrent huge agglomeration of horses from other properties (*p* = 0.3). In the buffy coat evaluation for hemoparasites, including *Trypanosoma evansi* included, all samples were negative.

### Viruses

**Fig 2:**
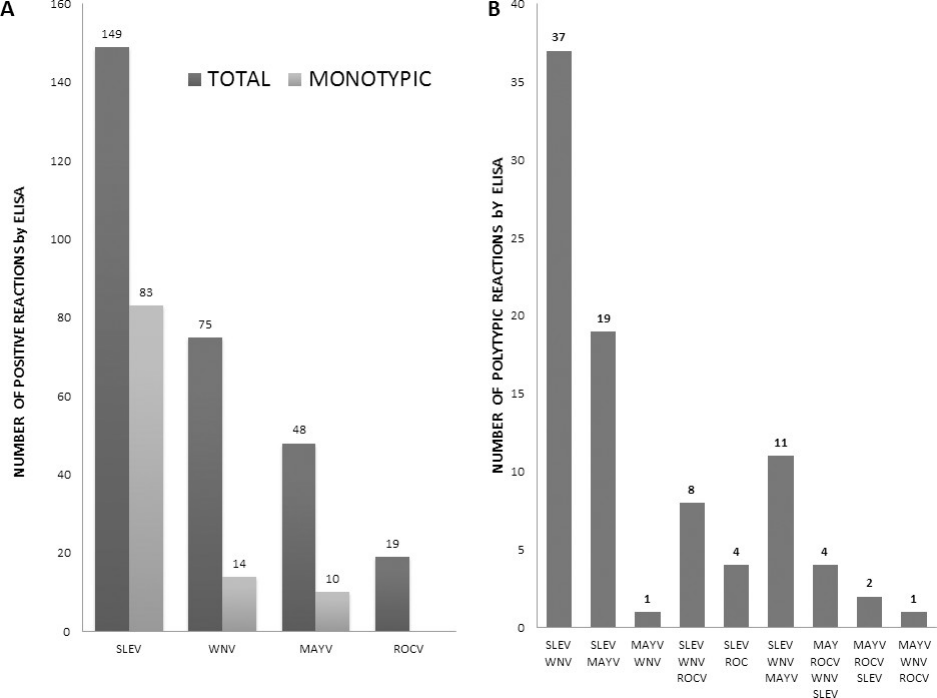
Positive ELISA assays for arboviruses in equines from the south of Roraima state. (A): Total positive results by ELISA and total monotypic (when reacting with only one of the viruses) reactions. (B): Polytypic (when reacting with two or more viruses) results for arboviruses by ELISA. SLEV: St. Louis encephalitis virus; WNV: West Nile virus; MAYV: Mayaro virus; ROCV: Rocio virus.

One hundred and forty-nine horses (70% seropositivity) had IgG antibodies to SLEV, 75 (35.2%) to WNV, 48 (22.5%) to MAYV and 19 (8.9%) to ROCV. Regarding flaviviruses, 97 samples presented a monotypic reaction (to only one virus) and were selected for confirmation by a virus-specific neutralization assay (PRNT_90_). Other sera presented polytypic reactivity (the same serum sample reacted to two or more viruses), and the most common association was SLEV-WNV in 37 of 87 sera samples (42.5%). All 19 horses with seropositivity test for ROCV by ELISA also were positive for another *Flavivirus* (Fig 2A). A total of 48 sera were positive for the *Alphavirus* MAYV, and curiously 38 of them were positive for at least additional *Flavivirus* (Fig 2B).

#### St. Louis encephalitis virus (SLEV)

A total of 92% (n = 76/83) of the monotypic positivity for SLEV were also positive in PRNT_90_, with titers ranging from 1:10 to 1:5120 (S1 Table). Regarding categories agglomeration and contact with dead or recovered horses the results were distributed in the following way: i) with contact 15 (1: 10–1:80), 29 (1:160–1:640), 0 (1:1280–1:5120), without contact 11 (1: 10–1:80), 18 (1:160–1:640), 3 (1:1280–1:5120); ii) with agglomeration 10 (1: 10–1:80), 22 (1:160–1:640), 2 (1:1280–1:5120), without agglomeration 16 (1: 10–1:80), 25 (1:160–1:640), 1 (1:1280–1:5120).

No difference in positivity was observed regarding contact of horses with dead or recovered horses from neurological symptoms (*p* = 0.2) and the habit to visit or dwell on farms where are a recurrent huge agglomeration of horses from other properties (*p* = 0.4). Among the 76 PRNT_90_ positive horses, 7.8% (n = 6) were also positive also for MAYV, and 41% (n = 31) were positive for *S*. *neurona*.

#### West Nile virus (WNV)

A total of 43% (n = 6/14) of horses displaying monotypic positivity for WNV were also positive by PRNT_90_, the highest titer was 1:80, being 1 (1:10), 2 (1:20), 1 (1:40) and 2 (1:80). Half of the WNV PRNT_90_ positive horses lived in properties located in the center of Rorainópolis the municipality with the most extensively human-modified landscape out of all the study area.

#### Mayaro virus (MAYV)

A total of 33% (n = 16/48) of horses were positive in PRNT_90_ for MAYV with low titers 12 (1:10), 3 (1:20) and 1 (1:40); six horses 4 (1:10) and 2 (1:20) were also positive for SLEV (S1 Table). No difference in positivity was observed regarding contact of horses with dead or recovered horses from neurological symptoms (*p* = 0.08) and the habit to visit or dwell on farms where are a recurrent huge agglomeration of horses from other properties (*p* = 0.4) (Table 1). Of the four recovered horses, two of them had coinfections by SLEV (PRNT_90_) and *S*. *neurona*, one was positive for SLEV and the other was positive for *S*. *neurona* (S1 Table).

## Discussion

Protozoan infection was investigated because neurological symptoms in horses may be related to these parasites, such as *Neospora hughesi*, *Trypanosoma evansi* and *S*. *neurona* [1, 33, 34]. Our results demonstrated that *Neospora* spp. and *Toxoplasma gondii* had low circulation, and *T*. *evansi* was absent among the studied horses since all horses tested negative for trypanosomiasis, and only two of them (0.9%) were positive for these coccidiosis. The infections by *Neospora* spp. are probably related to herd management, since the two infected horses derived from beef and dairy farms, were in close contact with cattle and dogs, a known risk factor for infection by *Neospora* spp. [35–38]. A high infection rate by *S*. *neurona* in our study provides evidence to suppose that in Roraima there are definitive hosts, probably opossums, maintaining the *S*. *neurona* biological cycle; since the variation in the seroprevalence has typically been attributed to climate and the density of competent hosts [2], our infection rate was lower than that observed in another region of the Amazon biome, in the Rondônia state, where among 192 horses, 84.4% were positive by recombinant ELISA assay [39]

South Roraima state presents some features, such as floodplain areas, primary forest and a small densely populated area that could enhance the dissemination and maintenance of arboviruses. We found at least two closely related flaviviruses that induced cross-reactive antibodies. The cross-reaction of antibodies between related viruses is a phenomenon commonly observed flaviviruses of the Japanese encephalitis serocomplex, as is the case for SLEV and WNV [40,41]. Horse sera were screened using a recombinant rDIII and rE2 antigen ELISA for flaviviruses and Mayaro, respectively. Monotypic *Flavivirus*-monotypic positive results were confirmed using a PRNT threshold of 90%, to optimize the virus-specific diagnosis.

In the present study, 23% of the horses (n = 49/213) presented positive results for more than one *Flavivirus* by ELISA, suggesting that one or more viruses of this genera infected horses in the area. Our data also suggest that cross-reactions occured in the sera of most of the studied horses. Cocirculation of more than one *Flavivirus* was detected on one farm only (F15), where the cocirculating viruses were probably SLEV and WNV. Considering that 92% (n = 76/83) of monotypic samples for SLEV by ELISA were confirmed by the PRNT, which is considered the gold standard for detecting neutralizing antibodies [41], it is unlikely that the low WNV titers by the PRNT were due to cross-reactivity between these two viruses; however, it does not rule out that this result could be due to another phylogenetically closely related virus. It is important to point out that the rDIII ELISA has not been evaluated for its sensitivity/specificity for IgG detection, meaning that we might have not detected some positive samples in the initial screening, and consequently, these samples were not evaluated by a specific PRNT. For a satisfactory accuracy, it would be necessary to analyze *Flavivirus* polytypic samples with ELISA screening. The rE2 ELISA for MAYV has been reported as having 100% sensitivity and approximately 80% specificity for IgG detection [31].

Considering the ELISA for SLEV, the studied horses presented an infection rate higher than the mean infection rate in the other five regions of Brazil (38.9%, n = 83/213 vs. 12.3%, n = 93/753 in monotypic horses) [42]. Our infection rate for SLEV based on the PRNT_90_, 35.6% (n = 76/213), is similar to infection rates previously described in the Central Region of Brazil (40% to 45%) [43]. In another serological survey of 1401 horses from five municipalities (four in the Amazonian region), the mean infection rate for SLEV was 50.9% [44]. Thus, the detection of horses infected by St. Louis encephalitis virus in our study confirms that this virus is enzootic in Roraima state.

We highlight a property (F1; n = 16) with a high infection rate by SLEV that also reported the deaths of many horses with neurological symptoms from 2014 to 2016. This farm had 1000 bovines, 150 sheep, 200 pigs, 200 ducks and 33 equines with free contact, and particularly, the horses there lived there in precarious conditions of hygiene. The same farm also reported many young ducks with neurological disease. Thus, SLEV, an avian zoonotic virus, could have been introduced into the area causing neurologic disease in ducks and horses.

WNV has been reported in equines in South America since 2006 [45]. In Brazil, there was a recent report of WNV isolation from a horse’s brain with neurological symptoms [46] in the Espírito Santo state, southeastern Brazil. An infection rate of 33.9% for SLEV, WNV and ROCV was detected with the rDIII antigen ELISA in 753 horses from three geographic regions of Brazil (southeast, northeast and central) [42,47], a similar result to the 35.2% observed in Roraima. Nevertheless, monotypic reactions to WNV were confirmed by the PRNT in only 11.4% (n = 9/79) of the animals whereas in Roraima, it was almost four-fold higher (43%; n = 6/14). All 19 Roraima horses with antibodies to ROCV also reacted to other flaviviruses preventing a confirmation of a previous infection by this virus.

In 2011 and 2014, horses in the midwestern region of Brazil were tested using a blocking ELISA followed by a confirmatory PRNT. In 2011, the infection rate for WNV was 3% and 3.2% in 2014 (based on the CDC positivity criterion) [48,49], both results are similar to those observed in Roraima (2.8%). In the present study, all six horses infected by WNV were derived from areas with a strong anthropogenic disturbance, a spatial distribution pattern similar to that found in the Central Region of Brazil [50]. Half of the horses were derived from the Rorainópolis municipality, which is the largest focus of human occupation in the south of the Roraima state (S1 Table and Fig 1). None of the infected horses travelled outside of Rorainópolis municipality, confirming the autochthony of these infections. Therefore, our results agree with those of other authors, suggesting that WNV has been circulated over the last decade in large areas of Brazil and infecting horses.

MAYV is an endemic virus in the Amazon region where it infects nonhuman primates [51], in our work a high proportion (33%) of the studied horses had neutralizing antibodies to MAYV, however PRNT _90_ values are quite low in these animals (≤ 1:40), suggesting possible cross-reaction with other alphaviruses. six horses presented antibodies to MAYV and SLEV suggesting that both viruses could cocirculate in the studied region.

Three out of the four recovered horses presented PRNT_90_ SLEV titers higher than 1:160, and two presented titers higher than 1:640 without positive results in ELISA screenings for other viruses. Infection by *S*. *neurona* was also detected in three of the four recovered horses, suggesting that SLEV (perhaps associated with a coinfection with *S*. *neurona*) may be responsible for neurological diseases in south Roraima horses.

## Conclusions

Horses were probably infected by WNV, suggesting that this virus reached this Amazonian region of Brazil. ROCV was not confirmed and is probably not circulating in the area. SLEV and *S*. *neurona* were found to individually and coinfect studied horses, including two of them that recovered from neurological symptoms.

Surveillance of new cases as well as virologic and protozoologic diagnoses are necessary to clarify the possible correlation of SLEV/*S*. *neurona* infections and the genesis of neurologic disease presented in Roraima horses.

## Supporting information

S1 Table Indirect serological diagnosis for arboviruses, in horses of the South of Roraima state. (XLSX)
